# Raman microscopy evaluation of the preventive effect of a modified orthodontic adhesive with silver nanoparticles on the formation of white spot lesions

**DOI:** 10.4317/jced.60531

**Published:** 2023-09-01

**Authors:** Marco Sánchez-Tito, José-Alberto Castañeda-Vía, Lidia-Yileng Tay

**Affiliations:** 1 Faculty of Health Sciences, Universidad Privada de Tacna, Tacna, Peru; 2 PhD Program in Stomatology, Universidad Peruana Cayetano Heredia, Lima, Peru; 3 PhD Program in Life Sciences, Universidad Peruana Cayetano Heredia, Lima, Peru; 4 Faculty of Stomatology, Universidad Peruana Cayetano Heredia, Lima, Peru

## Abstract

**Background:**

Silver nanoparticles (AgNPs) are an effective antibacterial agent, and their inclusion in orthodontic adhesives has been proposed to prevent white spot lesions (WSLs). The objective of this study was to evaluate by Raman microscopy the preventive effect of an orthodontic adhesive modified with AgNPs on the formation of WSLs.

**Material and Methods:**

AgNPs were added in four concentrations (0.05%, 0.1%, 0.5%, and 1%) to an orthodontic adhesive. Metal brackets were bonded with the experimental adhesives, and the specimens were subjected to a microbial model for caries induction. The preventive effect on the formation of WSLs was evaluated by Raman microscopy, considering the intensity peak of the phosphate ion at 960 cm-1. The acquisition mode was linear scanning in the most representative lesion area, with a length of 136µm. Intensity values expressed the relative amount of phosphate ions remaining in the lesion. Microphotographs were analyzed in the Image J program to assess the depth of the lesions.

**Results:**

Significant differences were observed between all groups (*P* <0.05). The addition of 1% AgNPs effectively maintained the relative amount of phosphate ions close to sound enamel values. Furthermore, as the concentration of AgNPs increased, the depth of the lesions decreased.

**Conclusions:**

AgNPs were effective in decreasing the formation of WSLs. At a higher concentration of AgNPs, a more significant preventive effect on the formation of WSLs results in a relative amount of phosphate ion close to sound enamel values.

** Key words:**White spot lesions, antibacterial, orthodontics, Raman microscopy, adhesive, Silver-nanoparticles.

## Introduction

The increased colonization of cariogenic bacteria and the consequent biofilm formation around the brackets during orthodontic treatment are predisposing factors for the formation of white spot lesions (WSLs) (1,2). The decrease in pH levels close to critical values and the amount of phosphate, calcium, and fluoride ions available in the oral environment have a direct impact on the formation of WSLs (3).

WSLs can reach a prevalence more significant than 60% in patients undergoing fixed orthodontic treatment (4,5). It is recognized that WSLs are more frequent in young patients with poor oral hygiene and prolonged treatments (6). In addition, these lesions significantly impact the patients’ esthetics (7).

Different strategies focused on the prevention and treatment of WSLs have been suggested by some authors. Regimens to improve oral hygiene (4), use of mouthwashes (8), fluoride varnishes (9), and sealants (10) around the brackets have been used to prevent the formation of WSLs. On the other hand, several antibacterial agents have been incorporated into orthodontic adhesives, such as quaternary ammonium compounds (11), monomers (12), metallic nanoparticles (13,14), among others; aiming to reduce bacterial growth and colonization around orthodontic brackets.

The use of silver nanoparticles (AgNPs) has been widely used in dentistry as an antibacterial agent. The antibacterial property of AgNPs is related to the size, shape, charge, and dose used (15,16). Some authors have tested the antibacterial properties of the incorporation of AgNPs into orthodontic bonding agents, showing that they are adequate to inhibit the growth of acidogenic bacteria (17,18), promoting a preventive effect on the formation of WSLs (19).

Previous studies evaluated the effect of adding AgNPs into orthodontic adhesives on the prevention of WSLs, employing methods such as microhardness test (20), changes in the metabolic activity of bacteria (17), and counting of colony-forming units (19,21). To date, no study has been conducted to evaluate the preventive effect of an orthodontic adhesive modified with AgNPs on the formation of WSLs using Raman microscopy. This method has been proposed as a sensitive and specific approach for evaluating enamel demineralization processes (22,23). The characterization of the demineralization lesions is carried out by comparing the changes in the number of minerals in the lesion concerning sound enamel (24).

In this sense, it is relevant to study the relative amount of phosphate ions present in the demineralization zones and the depth of the lesions as objective measurement of the preventive effect on the formation of WSLs. The objective of this study was to evaluate the preventive effect of an orthodontic adhesive modified with different concentrations of AgNPs on the formation of white spot lesions. In this study, we used Raman microscopy to characterize the demineralization lesions in the enamel.

## Material and Methods

This study was approved by the local ethics committee of the Universidad Peruana Cayetano Heredia (protocol No. 310-15-19). Sample size calculation was performed using G*Power 3.1.3 software (Heinrich Heine Universität, Düsseldorf, Germany) using an ANOVA test with fixed effects, with an effect size of 0.81, an α = 0.05, and a power of 95%, in accordance with a previous study (25). A total of Twenty-five teeth were calculated, five samples were randomly distributed in each group (n=5). Sound lower premolar teeth extracted for orthodontic reasons were collected from private clinics. The teeth were cleaned, and tissue remnants were removed with Gracey curettes. Then, the teeth were stored in distilled water at 4°C until use.

The experimental orthodontic adhesive was formulated by mixing a conventional light-cured orthodontic adhesive (Transbond XT; 3M Unitek, Monrovia, CA, USA) with different concentrations of AgNPs (US Research Nanomaterials, Houston, TX, USA, average particle size: 20 nm, purity: 99.99%) by wt/wt%. The detail of the amounts of material used is shown in Table 1. The manual mixture was made in a dark environment at room temperature (26) until a homogeneous consistency was achieved. The experimental adhesives were stored in black syringes to avoid light transmission.

**Table 1:**
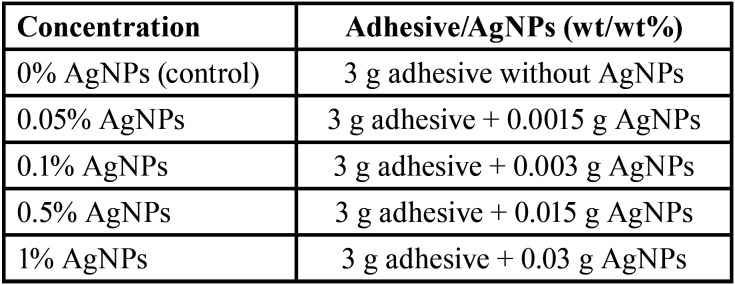
Composition of the experimental adhesives.

The teeth were embedded in silicon impression material (Zetaplus, Zhermack SpA, Italy) in PVC molds (15-mm inner diameter and 20-mm height) with the cementoenamel junction 2 mm above the silicon. The specimens were randomly assigned to five groups (n = 5) according to the concentration of AgNPs in the adhesive. Additionally, the conventional adhesive was used as a bonding agent for the control group. The buccal surface of the teeth was polished with pumice for 30 s and washed with distilled water. 37% phosphoric acid (Unitek Etching Gel, 3M, Monrovia, CA, USA) was applied for 30 s (17,27), washed with distilled water for 30 s, and air-dried. A thin primer layer (Transbond XT Primer; 3M Unitek, Monrovia, CA, USA) was applied and brushed on the enamel surface. Promptly, lower premolar metal brackets (Gemini, 3M Unitek, Monrovia, CA, USA) were bonded using the modified and conventional adhesives. The bracket was positioned parallel to the long axis of the tooth at the buccal surface (27). To standardize the thickness of the adhesive, a constant force of 300 gF was applied for 5 s with a dynamometer (Correx, Bern, Switzerland) (12). Excess resin was carefully removed with a scale, and the specimens were light-cured for 20 s on the mesial and distal side of the bracket (40 s in total) with a Led unit (Elipar DeepCure-L unit; 3M Unitek, Monrovia, CA, USA) at 450 nm wavelength, 1470 mW/cm2, with a constant distance of 3 mm (17,28).

A cariogenic solution was prepared by mixing 400 mL of distilled water with 14.8 g of BHI, 4 g glucose, 8 g sucrose, and 2 g yeast extract (29). The solution was autoclaved for 15 min at 121°C. Bacterial suspensions of *Streptococcus mutans* (ATCC® 25175TM, Manassas, VA, USA) and *Lactobacillus* acidophilus (ATCC® 4356TM, Manssas, VA, USA) were prepared using BHI and MRS as culture medium, respectively. The strain cultures were incubated for 7 h at 37°C under microaerophilic conditions (GaspakTM EZ Campy Container System). An aliquot of the suspensions was transferred to a test tube containing physiologic serum and adjusted to 0.5 McFarland Standard.

The teeth were removed from the molds, and the crowns and roots were covered with two layers of acid-resistant nail varnish (Maybelline New York, NY, US). An area of 2.5 mm x 2 mm was left in the bracket’s cervical region to allow the cariogenic solution’s effect (30). The teeth were individually placed in sterile conical FalconTM tubes, and 25 mL of the cariogenic solution and 25mL of the bacterial suspensions were added. The Falcon tubes were incubated for 9 days at 37°C. The culture medium was changed every 48 h without adding bacterial suspension.

The brackets were carefully debonded with Howe pliers, and the remaining adhesive was removed with an 18-blade bur (FG No. 118, Ortomundi, Londrina, Brazil). The complete removal of the adhesive was evaluated with a stereomicroscope at 20x magnification (AmScope SM20, United Scope LLC, USA). Under constant water irrigation, the teeth were cross-sectioned with a diamond disc (930D Jota, Switzerland) at low-speed rotation (Kavo 500 micromotor, Germany). In addition, a cut was made at 5 mm of the cementoenamel junction from the buccal face. Subsequently, one of the sections of the teeth was mounted on a PVC tube with an internal diameter of 15-mm and a height of 10-mm, with the cross-section down and was embedded with self-curing acrylic resin (VIPIflash, SP, Brazil). Once the polymerization process was completed, the teeth surface was manually polished with #600, #800, #1000, and #2000 sandpaper for 30 s each (28). Specimens were stored in distilled water until analysis.

For Raman microscopy, the changes in the intensity of the peak in the most powerful vibrational mode of the phosphate (PO43-) ions were observed at 960 cm-1, corresponding to a symmetrical stretching mode v1(PO43-) (25,31,32). Raman spectra were recorded using an Alpha 300 RA Confocal Raman microscopy system (WITec GmbH, Ulm, Germany). The excitation of the specimens was carried out with a 532 nm wavelength laser source with a power of 50 mW/cm2 and a diffraction grating of 300 lines/mm. The incident laser beam was focused on the specimen through a 20X Zeiss EC Epiplan objective. The acquisition mode was by linear scanning in the most representative area of the demineralization lesion, from the outer surface of the enamel (22,25), with a total length of 136µm, and detection every 5µm. The acquisition time was 10 s per measurement, with a total of 20 spectra of 0.5 s each, averaged at each point. The processing of the acquired spectra was performed using Project FIVE 5.9 software (WITec GmbH, Ulm, Germany).

The values obtained corresponding to the intensity of the peak v1(PO43-) recorded along the demineralization zone for each specimen were weighted percentage based on the intensity recorded for sound enamel, and the sigmoidal Boltzmann curve was used as the fit model. This procedure allowed to express the relative amount of phosphate (PO43-) ions that were remaining in the demineralization zones as a percentage.

**Figure 1:**
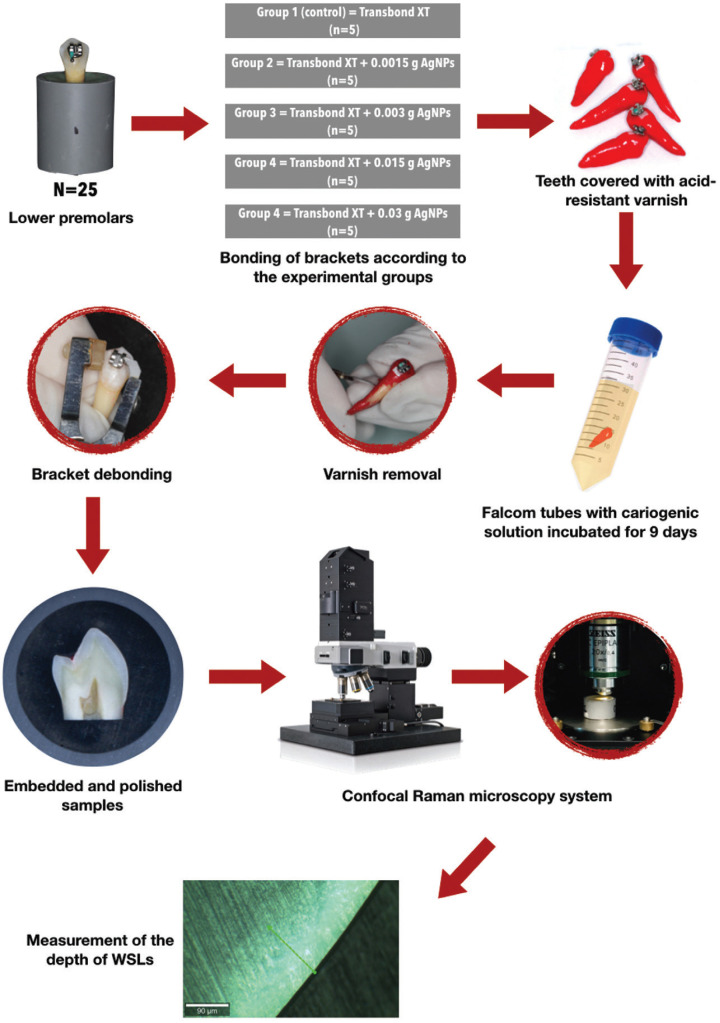
Flowchart of the methodology procedures used in this study.

Microphotographs obtained with the digital camera of the confocal Raman microscopy system were converted to JPG format and imported into the Image J program (Image J, 1.52v for Mac OS, US National Institutes of Health, Bethesda, Md). Microphotographs were converted to 8-bit grayscale, and brightness and contrast values were adjusted for better visualization. The scale was adjusted to pixels/µm to measure the demineralization zones’ length concerning the scale contained in the microphotographs. The depth of the demineralization lesion was measured with the freehand straight line tool, making 10 linear measurements on the extent of the lesion from the outer surface to the most extensive observable depth of the lesion. Figure 1 represents the flowchart of the methodology used in the present study.

Data normal distribution and homoscedasticity were assessed by Shapiro-Wilk and Levene’s test, respectively. To compare the relative amount of phosphate ions and the length of the WSLs, Kruskal Wallis and Dunn’s post hoc test for multiple comparisons were selected. The epsilon squared coefficient was used to estimate the effect size. Spearman’s correlation was used to verify the relationship between the concentration of AgNPs in the adhesive and the depth of the demineralization lesion. Stata 16.0 for Mac OS (StataCorp LP, College Station, TX, USA) was used to perform all statical tests, and the significance level was set at α = 5%.

## Results

The relative amount of phosphate (PO43-) ions in the lesion of each specimen was compared to the sound enamel. The results showed significant differences between the groups (*P* <0.05); (Table 2). These results indicate that the relative amount of phosphate ions in the specimens with 1% AgNPs remained close to 70.41% compared with the values measured in the sound enamel. Additionally, the effect size (ER2 = 0.368) indicates that the 36.8% variability in the intensity of the phosphate ions can be attributed to the concentrations of AgNPs.

**Table 2:**
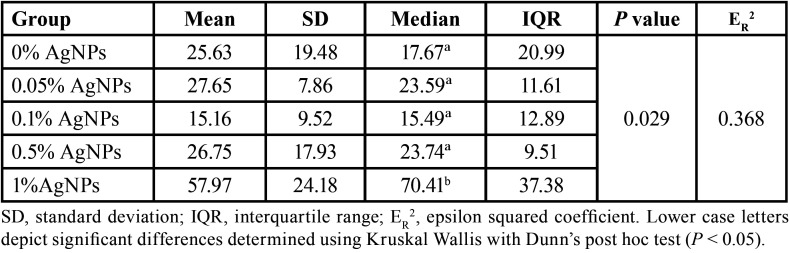
Comparison of the relative amount (%) of the phosphate ions between the experimental groups.

**Figure 2:**
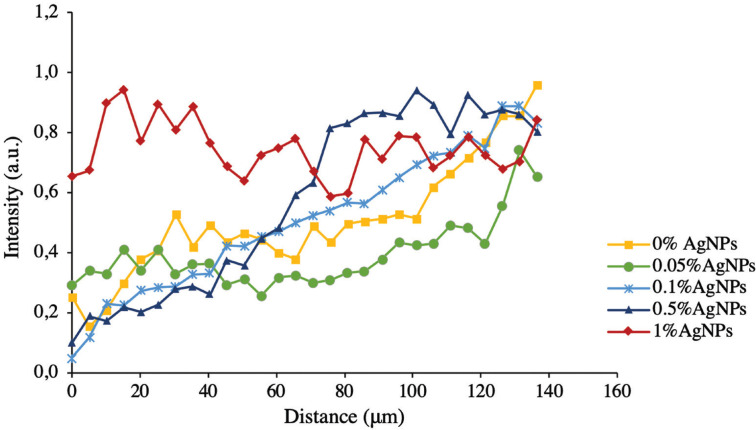
Relative intensity variation of the 961 cm-1 peak of the phosphate ion between the experimental groups.

Figure 2 shows the variation of the phosphate peak in the vibrational band 960 cm-1 of the experimental groups. The graph expresses the normalized relative intensity of the phosphate ions with respect to the linear distance of 136µm from the enamel surface. For the control group and groups with 0.05%, 0.1%, and 0.5% of AgNPs, the intensity of the phosphate ions decreased from the outer enamel surface and remained low up to a distance of 60-80µm. The group with 1% AgNPs showed a quick intensity recovery (20µm). At a distance of 160µm, all groups showed a recovery in the phosphate ions intensities close to sound enamel.

**Figure 3:**
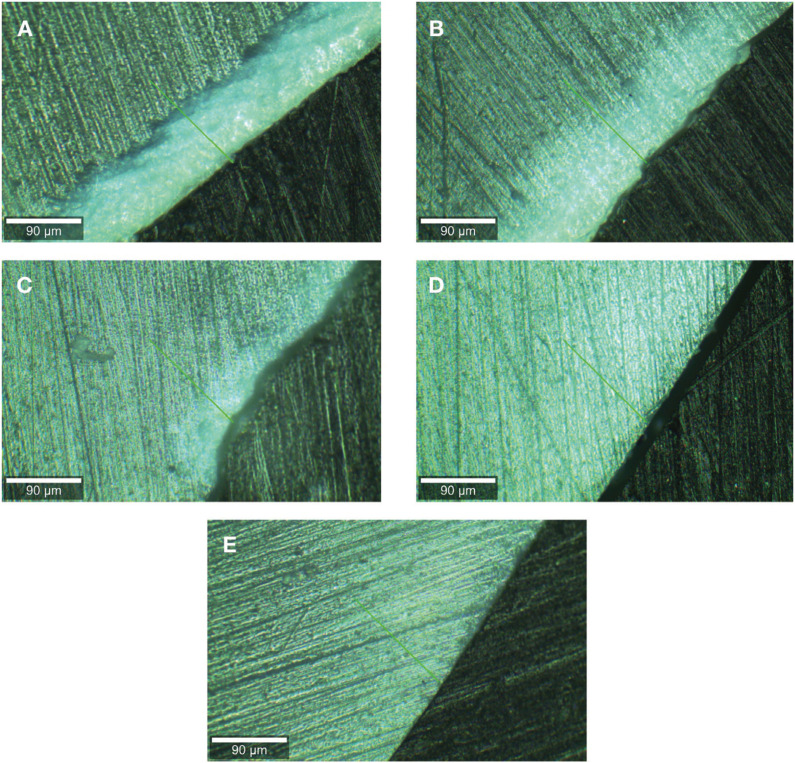
Detail of the formation of white spot lesions in a representative sample of each group according: (A) control group 0% AgNPs; (B) 0.05% AgNPs; (C) 0.1% AgNPs; (D) 0.5% AgNPs; (E) 1% AgNPs.

Table 3 shows that the depth values of the lesions of the groups with 0.05% and 0.1% of AgNPs were significantly higher than those registered for the groups with 0.5% and 1% of AgNPs (*P* <0.05). The estimated effect size (ER2 = 0.728); indicates that 72.8% of the variability in the depth of the lesion is attributed to the concentration of AgNPs. Moreover, there was a significant negative correlation between the amount of AgNPs added to the orthodontic adhesive and the depth of the demineralization zones (rho = -0.851, *P* <0.001), indicating that at a higher concentration of AgNPs in the adhesive, a lower depth of the demineralization lesion was observed. Figure 3 shows representative microphotographs of the experimental groups.

**Table 3:**
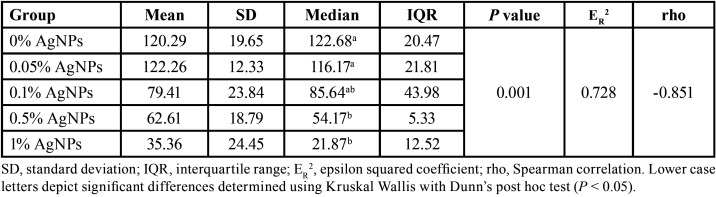
Depth of the demineralization zone between the groups (µm).

## Discussion

One of the most undesired side effects of orthodontic treatment is the formation of areas of enamel demineralization around the brackets in patients with poor oral hygiene. Recently, several studies have proposed incorporating antibacterial agents into orthodontic adhesives to prevent the formation of WSLs (11-14). These kinds of lesions are the result of changes in the chemical composition of the enamel when it is exposed to cariogenic factors (1,3). Therefore, in this study, AgNPs were added to an orthodontic adhesive as an antibacterial agent, and their preventive effect on the formation of WSLs was evaluated with a spectroscopy method.

Raman microscopy has been proposed as a sensitive and highly selective method for the characterization of enamel demineralization processes, allowing both quantitative and qualitative analysis (22,23). Quantitative chemical analysis performed by Raman microscopy is based on peak intensity/peak area ratio. The characterization of the structural components of the demineralization lesions is carried out by comparing the changes in the number of minerals contained in the lesion concerning sound enamel (24,33). The phosphate (PO43-) ion presents four vibrational modes, and the most substantial peak is observed at 960 cm-1, which is attributed to the symmetric stretching mode (v1), and it is used to assess the progression and intensity of the lesions (34).

In this study, the addition of different concentrations of AgNPs into the orthodontic adhesive showed variation in the relative amount of phosphate ions compared with sound enamel. The adhesive with 1% AgNPs maintained the relative amount of phosphate (PO43-) ions close to 70.41% in relation to sound enamel. In addition, the low intensity of the phosphate (PO43-) ions observed in the lesions of the control group (without AgNPs) indicates that their phosphate content was considerably lower (17.67%). These results can be explained since the intensity of the phosphate ions peaks is higher in sound enamel compared to the demineralized areas (22). In a study conducted by Milly *et al*. (35) where the effect of enamel remineralizing products was evaluated, a high reduction in the intensity peak of the phosphate ions in the demineralized enamel was observed, and the lesion presented 40% of phosphate peak intensity compared with sound enamel.

The drop in the relative intensity of the phosphate ions observed in the groups with 0.05%, 0.1%, and 0.5% AgNPs and control extended to approximately 60-80 µm from the outer surface of the enamel. This result is consistent with a previous report (35). The addition of 1% of AgNPs into the orthodontic adhesive seems to provide a preventive effect on the formation of WSLs since the intensity of the phosphate ions recovered rapidly (20µm). In all cases, the pattern showed that the phosphate ion intensities reached values close to sound enamel at a distance of 160µm. In addition, when the depth of the demineralization lesions was evaluated, the results showed a high negative correlation, indicating that at a higher concentration of AgNPs in the adhesive, a lower depth of the demineralization lesion was observed.

Mohanty *et al*. (22) showed that in incipient caries lesions, phosphate ion intensities were higher on the enamel surface but decreased rapidly on the subsurface and remained constant down to a depth of 100-120µm until reaching values close to sound enamel at a distance of 200µm. Al-Obadi *et al*. (25) evaluated the formation of artificial and natural WSLs through Raman microscopy. Their results showed that linear scanning identified a phosphate ion signal on the outer surface of enamel in all their samples, indicating the presence of an intact enamel surface, followed by a severe deflection of the phosphate ions peak in the area corresponding to the lesion. In addition, at a greater distance in the enamel, the intensity peak of the phosphate ions approached that of the sound enamel.

This pattern of intensity of the phosphate ions on the enamel surface differs from the results found in our study, where the intensity of the phosphate ions decreases directly from the outermost surface of the enamel. These differences could be explained by the use of different models for the induction of WSLs. In the case of the cited studies, chemical methods based on acidic solutions were used, while in this study, a bacterial model reported previously was used for 9 days (29). Bacterial models allow the study of the effect of antibacterial agents and have been used to evaluate the inhibition of demineralization of dental tissues (36).

The preventive effect on the formation of WSLs observed in this study can be attributed to the amount of AgNPs added to the orthodontic adhesive. It has been previously reported that a concentration of 1% AgNPs promotes a significant antibacterial effect without affecting the mechanical properties of the adhesives (14,19,26). The antibacterial mechanism of AgNPs has not been fully elucidated, but it has been hypothesized that it is related to the positive charge of the silver ions, which produces an electrostatic attraction with the negative charge of the bacterial membrane; allowing the entry of silver ions into the cytoplasm and degrading the function of proteins and enzymes, consequently leading to cell death (16,37). Other factors such as the size and shape of the nanoparticles also have an effect on the antibacterial properties (38). It has been shown that smaller AgNPs exert a more significant antibacterial effect against various bacterial species (39). In this study, AgNPs of 20 nm diameter were used, and their effectiveness was previously demonstrated in reducing the formation of WSLs (19). A small particle size offers a large surface area, allowing a greater release of silver ions and consequently a greater antibacterial effect (40).

In this study, the preventive effect on the formation of WSLs of an orthodontic adhesive modified with AgNPs was studied by Raman microscopy, considering the limitations of an *in vitro* model, where the conditions for the formation of WSLs are not entirely similar to those of the oral environment. Further investigation is necessary to evaluate the clinical application of modified orthodontic adhesives with antibacterial agents from the premise of providing preventive methods that help to reduce the negative impact of WSLs.

## Conclusions

AgNPs were effective in decreasing the formation of WSLs. At a higher concentration of AgNPs, a more significant preventive effect on the formation of WSLs expressed in a relative amount of phosphate (PO43-) ions closer to that of sound enamel. In addition, a higher concentration of AgNPs was observed to be related to a lower depth of the lesion.
